# Establishing baseline criteria of cardio-ankle vascular index as a new indicator of arteriosclerosis: a cross-sectional study

**DOI:** 10.1186/1471-2261-11-51

**Published:** 2011-08-10

**Authors:** Tsukasa Namekata, Kenji Suzuki, Norio Ishizuka, Kohji Shirai

**Affiliations:** 1 Pacific Rim Disease Prevention Center, P.O.Box 25444, Seattle, WA 98165-2344, USA; 2 Department of Health Services, School of Public Health, University of Washington, Box 357660, Seattle, WA 98195-7660, USA; 3 Japan Health Promotion Foundation, 1-24-4 Ebisu, Shibuya-ku, Tokyo, Japan; 4 Department of Internal Medicine, Sakura Hospital Medical Center, Toho University, 546-1 Shizu, Sakura-shi, Chiba Prefecture, Japan

## Abstract

**Background:**

A cardio-ankle vascular index (CAVI) has been developed to represent the extent of arteriosclerosis throughout the aorta, femoral artery and tibial artery independent of blood pressure. To practically use CAVI as a diagnostic tool for determining the extent of arteriosclerosis, our study objectives were (1) to establish the baseline CAVI scores by age and gender among cardiovascular disease (CVD) risk-free persons, (2) to compare CAVI scores between genders to test the hypothesis that the extent of arteriosclerosis in men is greater than in women, and (3) to compare CAVI scores between the CVD risk-free group and the CVD high-risk group in order to test the hypothesis that the extent of arteriosclerosis in the CVD high-risk group is greater than in the CVD risk-free group.

**Methods:**

Study subjects were 32,627 urban residents 20-74 years of age who participated in CVD screening in Japan during 2004-2006. A new device (model VaSera VS-1000) was used to measure CAVI scores. At the time of screening, CVD high-risk persons were defined as those having any clinical abnormalities of CVD, and CVD risk-free persons were defined as those without any clinical abnormalities of CVD. Age-specific average CAVI scores were compared between genders and between the CVD risk-free group and the CVD high-risk group. Student's t-test using two independent samples was applied to a comparison of means between two groups.

**Results:**

Average age-specific baseline scores of CAVI in the CVD risk-free group linearly increased in both genders as their age increased. Average age-specific baseline scores of CAVI in the CVD risk-free group were significantly greater among men than among women. Average age-specific baseline scores of CAVI in the CVD risk-free group were significantly smaller than those in the CVD high-risk group in both genders after 40 years of age.

**Conclusions:**

The baseline CAVI scores from the CVD risk-free group are useful for future studies as control values. The CAVI method is a useful tool to screen persons with moderate to advanced levels of arteriosclerosis.

## Background

One leading cause of premature deaths in industrialized nations is cardiovascular disease including coronary heart disease (CHD), an atherosclerosis-related disease. In 2005, the CHD death rates (per 100,000 persons) were 159.0 for US males, which was 2.3 times higher than for Japanese males (68.1), and 142.0 for US females, which was 2.7 times higher than for Japanese females (53.5) [1,2]. Thus, there is a great need to prevent CHD incidence as well as mortality in the US. One approach is to identify persons with moderately advanced state of arteriosclerosis and provide recommendations for improving their lifestyle and diet. Japan has been taking such an approach for the past few decades and successfully kept CHD mortality low [2].

One method to quantitatively estimate the extent of arteriosclerosis is the use of the pulse wave velocity (PWV). The idea on the association of PWV with arteriosclerosis is traced back to an experiment using artificial blood vessels conducted by Moens in 1878 [3]. Then, Bramwell and colleagues showed that PWV depends on the modulus of arterial volume elasticity by experiments in 1922-23 [3-7]. Their experimental results have been a basis for the development of the measurement device PWV-200 (Fukuda-Denshi Co., Tokyo) which measures PWV propagating through the aorta (thorax, abdomen, and part of common iliac artery) from the aortic valve to the femoral pulsation point, as described by Hasegawa in 1970 [8]. Because PWV is highly correlated with diastolic blood pressure, Hasegawa developed a nomogram showing the association between diastolic blood pressure and PWV. He proposed an adjustment to any measured PWV values at 80 mmHg. As a result, such an adjustment was built into the PWV-200 machine. This is an important step allowing clinicians and researchers to compare PWV values between individuals and between populations. Namekata et al. conducted cardiovascular disease prevention screening in Seattle and found that PWV was positively and significantly associated with aging (≥ 60 years of age), hypertension, diabetes, the ratio of total cholesterol to high density lipoprotein cholesterol, ex-smokers and negatively and significantly with alcohol consumption among Japanese Americans [9]. In addition, they had similar findings among Japanese urban workers [10].

To overcome some problems associated with PWV-200 (i.e., technical difficulty in the method for measuring PWV), the cardio-ankle vascular index (CAVI) was developed as a new indicator of arteriosclerosis in 2004 [11]. CAVI quantitatively reflects arteriosclerosis of the aorta, femoral and tibial arteries based on Bramwell-Hill's equation [3] and stiffness parameter [12] which is allowed to be converted from PWV propagating from the aortic valve to ankle. Some researchers proposed to use CAVI scores as an indicator of atherosclerosis. Nakamura et al. found a strong association of CAVI with the presence of severity of coronary atherosclerosis based on their ordinal logistic regression analysis [13]. Kadota et al. suggested the use of CAVI as a screening tool for atherosclerosis based on their findings from the general population study of 1,014 adults showing strong significant associations of CAVI scores with carotid intima-media thickness and with homocysteine after adjustment for age and sex [14]. Thus, it is considered that CAVI scores reflect arterial stiffness, atherosclerosis and arteriosclerosis of which conditions are overlapping and inseparable. We use CAVI to represent the extent of arteriosclerosis in this paper but it is inclusive of arterial stiffness and atherosclerosis.

To practically use CAVI as a diagnostic tool for determining the extent of arteriosclerosis, our study objectives are (1) to establish the baseline CAVI scores by age and gender among cardiovascular disease (CVD) risk-free persons, (2) to compare CAVI scores between genders to test the hypothesis that the extent of arteriosclerosis in men is greater than in women, and (3) to compare CAVI scores between the CVD risk-free group and the CVD high-risk group to test the hypothesis that the extent of arteriosclerosis in the CVD high-risk group is greater than in the CVD risk-free group.

## Methods

### Study Subjects

Subjects for the study were recruited through the screening program at Japan Health Promotion Foundation which has been conducting cardiovascular disease and cancer screening throughout major cities of Japan. Subjects were company employees and their family members: 16,661 men and 15,966 women between 20 and 74 years of age (see Table 1) after excluding persons with history of heart disease, hypertension, stroke, diabetes, nephritis, and gout. The proportion of CVD risk-free subjects to all subjects decreases as age advances (both genders combined): 45.4% for 20-29 years of age, 30.1% for 30-39 years of age, 18.7% for 40-49 years of age, 9.7% for 50-59 years of age, 6.9% for 60-69 years of age, and 3.7% (or only 36 CVD risk-free subjects out of 979 subjects) for 70-74 years of age.

**Table 1:** Subjects by age and sex

	All subjects		CVD risk-free subjects	
	
Age	Males	Females	Males	Females
20-29	1214	949	455	526
30-39	4008	3243	877	1307
40-49	3880	4111	421	1077
50-59	4619	5653	306	690
60-69	2319	1654	155	119
70-74	623	356	25	11

Total	16661	15966	2239	3730

The study was approved by the Institutional Review Board and all subjects gave their consent to participate in the study.

### Measuring Cardio-Ankle Vascular Index

**Figure 1:**
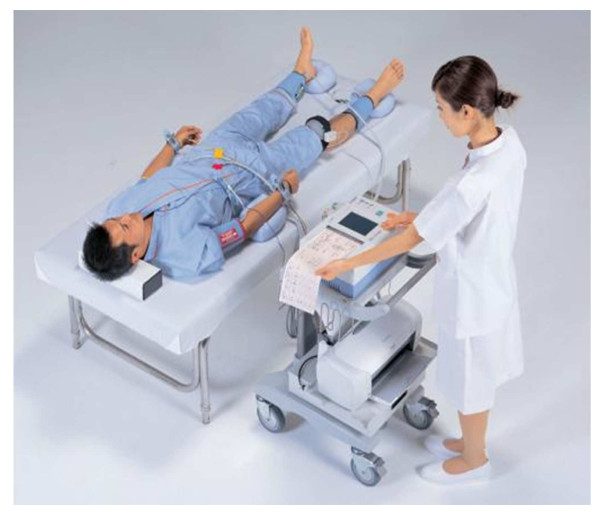
**Demonstration of CAVI measurement by VaSera VS-1000**.

CAVI, a stiffness and arteriosclerosis indicator of thorax, abdomen, common iliac, femoral and tibial arteries, is measured by VaSera VS-1000 manufactured by Fukuda-Denshi Company, LTD (Tokyo, Japan), as shown in Figure 1. This device is a new version of PWV-200. It is significantly improved as it achieved 3.8% of the average coefficient of variation among five repeated measurements of CAVI for each of the 22 subjects [11] showing that its operation is less dependent on a technician's skill. Furthermore, CAVI scores were not changed but brachial-ankle PWV values were significantly changed when both systolic and diastolic blood pressure of 12 healthy volunteer men was significantly changed after metoprolol (80 mg) was administered [15]. This suggests that CAVI is not affected by blood pressure at the time of measuring.

**Figure 2:**
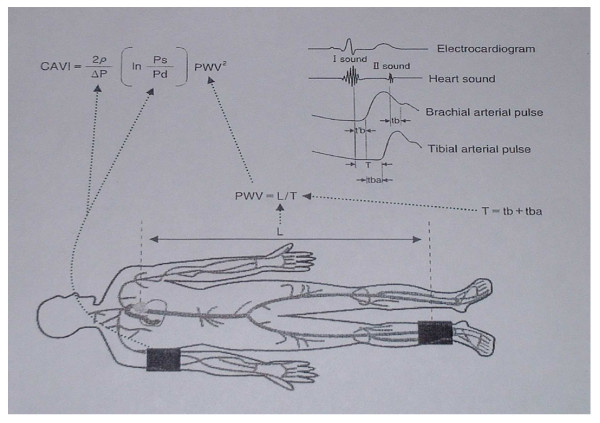
**Illustration of CAVI measurement**.

The method to measure CAVI is illustrated in Figure 2. A subject is placed in supine position and electrocardiogram and heart sound are monitored. PWV between heart and ankle is obtained by L/T where L is the distance from the aortic valve to the ankle, and T is the time during which PWV propagates from the aortic valve to the ankle (or the sum of tb and tba in place of t'b and tba, because t'b and tb are theoretically equal: tba is the time between the rise of the brachial pulse wave and the rise of the ankle pulse wave, tb is the time between the aortic valve's closing sound and the notch of the brachial pulse wave, and t'b is the time between the aortic valve's opening sound and the rise of the brachial pulse wave) [11].

The scale conversion from PWV to CAVI is performed by the following formula:

where P_s _and P_d _are systolic and diastolic blood pressure values, respectively, PWV is the pulse wave velocity between heart and ankle, ΔP is P_s_-P_d_, ρ is blood density, and a and b are constants. This equation was derived from Bramwell-Hill's equation [3] and stiffness parameter [12]. Scale conversion constants are determined so as to match CAVI with PWV by Hasegawa's method [8]. All these measurements and calculations are automatically made in VaSera VS-1000. More theoretical details of CAVI method are available elsewhere [11].

### Clinical Criteria for Selecting CVD Risk-Free Persons and CVD High-Risk Persons

Blood was drawn from the subjects after a 12 hour-fast. The following measurements were made: total cholesterol (TC), triglycerides (TG), creatinine (Cre) by enzymatic assay; high density lipoprotein cholesterol (HDL-C) by modified enzymatic method; uric acid by uricase peroxides method, glucose by hexokinase glucose-6-phosphate dehydrogenate assay, glyco-hemoglobin A1c (HbA1c) by latex agglutination, and white blood cells (WBC) by direct current detection method. To identify subjects with ischemic changes, outputs from electrocardiogram were classified by Minnesota code [16] which has been internationally and uniformly used in the epidemiology setting. Retinal photographs of the right eye were taken by non-mydriatic retinal camera (Canon Co., Tokyo, Japan) to identify subjects with abnormal changes in retinal arteries by using Scheie's classification method [17].

The criteria to select subjects for the CVD risk-free group and for the CVD high-risk group were based on the guidelines established by Japan Atherosclerosis Society and Japan Society of Hypertension [18-21]. The CVD risk-free persons were defined as those meeting the following clinical criteria at the time of screening:

• blood pressure: systolic blood pressure(SBP) ≤ 139 mmHg and diastolic blood pressure (DBP) ≤ 89 mmHg;

• serum lipids: TC ≤ 219 mg/dL, HDL-C = 40-99 mg/dL and TG ≤ 149 mg/dL;

• serum glucose: glucose ≤ 109 mg/dL and HbA1c ≤ 5.8%;

• renal function: creatinine: male ≤ 1.10 mg/dL, female ≤ 0.80 mg/dL and uric-acid ≤ 7.0 mg/dL for both genders;

• white blood cells: 3.2-8.5 × 10^3^/μL;

• electrocardiogram: excluding persons with 1-1-1 to1-3-6, 3-1 to 3-3, 4-1 to 4-4, 5-1 to 5-5, and 9-2; and

• retinal artery changes: no arteriolar sclerotic change and no hypertensive change.

The CVD high-risk persons were defined as those who fall in one or more following groups of clinical abnormalities at the time of screening:

• borderline hypertension group: SBP:140-159 mmHg, and/or DBP:90-99 mmHg;

• hypertension group: SBP ≥ 160 mmHg, and/or DBP ≥ 100 mmHg;

• abnormal lipid metabolism group: TC ≥ 240 mg/dL, TG ≥ 250 mg/dL, and/or HDL-C ≤ 34 mg/dL;

• borderline high-glucose group: serum glucose 110-125 mg/dL and/or HbA1c 5.9-6.1%;

• hyperglycemia group: serum glucose ≥ 126 mg/dL and/or HbA1c ≥ 6.2%;

• ischemic change group: 1-1-1 to 1-1-3 (abnormal Q wave), and/or 4-1 to 4-3 (ischemic change); and

• arteriolar sclerotic change group: sclerotic change ≥ II in Scheie's method.

### Statistics Methods

In addition to the use of descriptive statistics, Student's t-test using two independent samples was applied to a comparison of means between two groups and p < 0.05 was considered statistically significant. Statistical Packages for Social Sciences version 16 was used for data analysis.

## Results

As shown in Table 1, there were 2,239 men and 3,730 women who were free from clinical CVD abnormalities. Table 2 represents age-specific means and standard deviations of the baseline CAVI scores from the CVD risk-free group by age and gender. Age-specific average CAVI scores became higher in both genders as their age advanced and 0.22-0.66 of increment was added to the average CAVI score as the age increased to every 10 years.

**Table 2:** Comparison of average cardio-ankle vascular index (CAVI) scores of CVD risk-free subjects by age and gender

	Males		Females		t-value	p-value
			
Age	Mean	SD	Mean	SD		
20-29	6.69	0.70	6.57	0.66	2.89	p = 0.005
30-39	7.12	0.68	6.79	0.63	5.23	p < 0.001
40-49	7.59	0.70	7.29	0.66	7.82	p < 0.001
50-59	8.07	0.76	7.82	0.70	4.97	p < 0.001
60-69	8.73	0.81	8.26	0.72	4.95	p < 0.001
70-74	9.35	1.00	8.71	0.75	1.88	p = 0.071

Note: SD indicates standard deviation.

**Figure 3:**
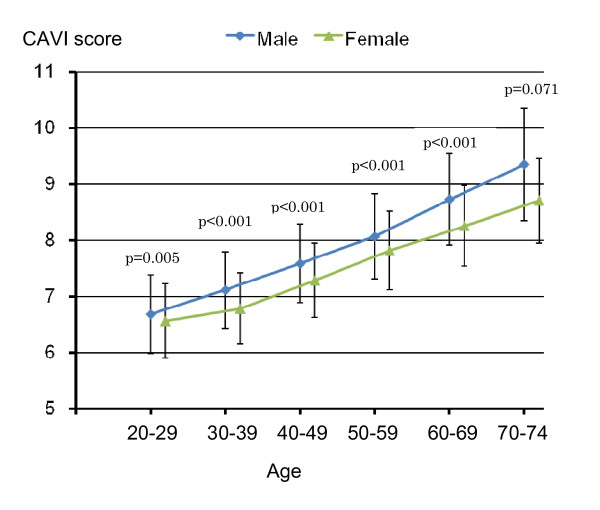
**Differences in average CAVI scores by age between males (blue line) and females (green line) among CVD risk-free individuals based on results shown in Table 2 (Vertical bars indicate standard deviation**.).

Figure 3 shows a comparison of age-specific average CAVI scores between genders. It is observed that average CAVI scores at each age-interval were significantly greater for men than for women with a borderline significance for 70-74 years of age (p = 0.071), and that men's CAVI scores were about 5 years ahead of women's between 30 and 60 years of age and even 10 years ahead of women's after 60 years of age.

Tables 3 and 4 show a comparison of average CAVI scores between the CVD risk-free group and each CVD high-risk group by age among men and among women, respectively. There were not enough cases of all CVD high-risk groups under 30 years of age for comparisons and of some CVD high-risk groups 30-39 years of age. Most average CAVI scores from each of the CVD high-risk groups were significantly higher than those from the CVD risk-free groups with one exception: the average CAVI score (6.95) of the hypercholesterolemia and hypertriglyceridemia group for men 30-39 years of age was significantly smaller than that (7.12) of the CVD risk-free group for the same gender and age-bracket (p = 0.021). On one hand, age-specific average CAVI scores of the hypertension group were significantly greater than those of the CVD risk-free group after 30 years of age among men but after 40 years of age among women. On the other hand, age-specific average CAVI scores of the hypercholesterolemia and hypertriglyceridemia group, the hyperglycemia group, the ischemic changes group, and the retinal artery changes group were significantly greater for both men and women after 40 years of age compared to those of the CVD risk-free group with exceptions of non-significance in the hypercholesterolemia and hypertriglyceridemia group of men 70-74 years of age (p = 0.106), in the hyperglycemia group of women 40-49 years of age (p = 0.093), and in the ischemic changes group and the retinal artery changes group of women 70-74 years of age (p = 0.052 and p = 0.071, respectively).

**Table 3:** Comparison of average CAVI scores between CVD risk-free group and CVD high-risk groups for males

Age	20-29	30-39	40-49	50-59	60-69	70-75
CVD risk-free group						
Mean	6.69	7.12	7.59	8.06	8.73	9.35
SD	0.70	0.68	0.70	0.76	0.81	1.00
Hypertension group						
Mean	-	7.43	7.86	8.47	9.12	9.84
SD		0.86	0.87	1.01	1.12	1.15
t-value		4.24	4.75	6.51	4.06	2.07
p-value		p < 0.001	p < 0.001	p < 0.001	p < 0.001	p = 0.041
Hypercholesterolemia & Hypertriglyceridemia group						
Mean	-	6.95	7.74	8.42	8.97	9.71
SD		0.84	0.86	0.95	0.91	0.82
t-value		-2.39	2.33	5.05	2.34	1.62
p-value		p = 0.021	p = 0.023	p < 0.001	p = 0.022	P = 0.106
Hyperglycemia group						
Mean	-	7.25	7.76	8.68	9.41	10.01
SD		0.88	0.82	0.98	1.65	1.40
t-value		1.14	2.31	8.80	6.94	2.19
p-value		p = 0.123	p = 0.034	p < 0.001	p < 0.001	p = 0.042
Ischemic changes group						
Mean	-	-	7.81	8.79	9.29	9.97
SD			0.70	1.12	0.87	1.31
t-value			2.29	8.36	5.43	2.14
p-value			p = 0.033	p < 0.001	p < 0.001	p = 0.043
Retinal artery changes group						
Mean	-	-	8.09	8.77	9.16	9.97
SD			0.77	1.25	1.10	1.14
t-value			2.60	6.69	3.77	2.38
p-value			p = 0.014	p < 0.001	p < 0.001	p = 0.022
All high-risk groups combined						
Mean	6.39	7.18	7.79	8.49	9.12	9.80
SD	0.69	0.85	0.85	0.98	1.05	1.14
t-value	-1.87	1.26	3.96	7.20	4.48	1.93
p-value	p = 0.061	p = 0.209	p < 0.001	p < 0.001	p < 0.001	p = 0.054

Note: SD is standard deviation and - indicates that the number of cases was too small to obtain any meaningful results.

**Table 4:** Comparison of average CAVI scores between CVD risk-free group and CVD high-risk groups for females

Age	20-29	30-39	40-49	50-59	60-69	70-75
CVD risk-free group						
Mean	6.57	6.97	7.29	7.82	8.26	8.71
SD	0.66	0.63	0.66	0.70	0.72	0.74
Hypertension group						
Mean	-	7.02	7.73	8.16	8.89	9.46
SD		0.74	1.02	0.84	0.96	1.02
t-value		0.40	6.85	8.22	6.44	2.38
p-value		p = 0.346	p < 0.001	p < 0.001	p < 0.001	p = 0.023
Hypercholesterolemia & Hypertriglyceridemia group						
Mean	-	6.97	7.68	8.00	8.77	9.26
SD		0.78	1.21	0.78	0.90	0.74
t-value		-0.03	4.12	3.82	5.06	2.28
p-value		p = 0.914	p < 0.001	p < 0.001	p < 0.001	p = 0.034
Hyperglycemia group						
Mean	-	-	7.47	8.16	9.09	9.77
SD			0.86	0.74	1.04	0.79
t-value			1.74	5.23	6.57	3.69
p-value			p = 0.093	p < 0.001	p < 0.001	p < 0.001
Ischemic changes group						
Mean	-	-	7.49	8.10	8.75	9.39
SD			0.82	0.82	0.88	1.02
t-value			2.95	5.71	4.69	2.07
p-value			p = 0.004	p < 0.001	p < 0.001	p = 0.052
Retinal artery changes group						
Mean	-	-	8.03	8.34	9.36	9.32
SD			0.89	0.86	1.11	0.87
t-value			3.97	5.24	7.37	1.92
p-value			p < 0.001	p < 0.001	p < 0.001	p = 0.071
All high-risk groups combined						
Mean	6.88	6.93	7.58	8.12	8.81	9.34
SD	0.42	0.76	0.91	0.81	0.96	0.99
t-value	1.48	-0.56	6.25	8.05	5.96	2.09
p-value	p = 0.138	p = 0.578	p < 0.001	p < 0.001	p < 0.001	p = 0.038

Note: SD is standard deviation and - indicates that the number of cases was too small to obtain any meaningful results.

**Figure 4:**
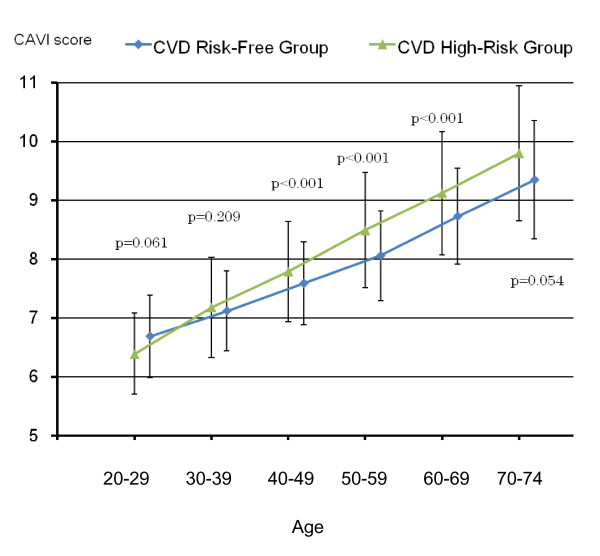
**Differences in average CAVI scores by age between the CVD risk-free group (blue line) and the CVD high-risk group (green line) for males based on results shown in Table 3 (Vertical bars indicate standard deviation**.)

**Figure 5:**
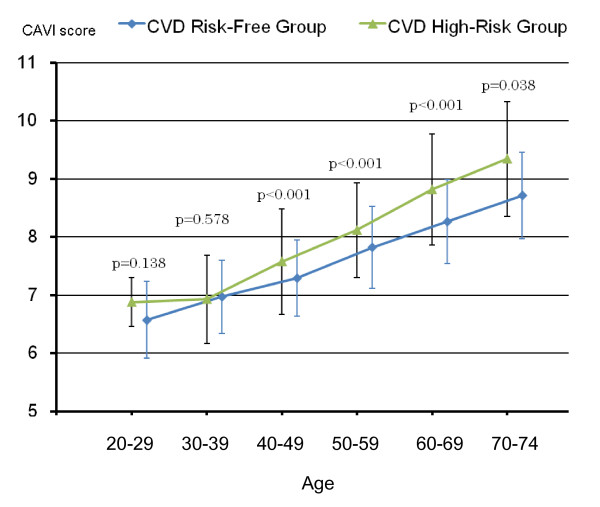
**Differences in average CAVI scores by age between the CVD risk-free group (blue line) and the CVD high-risk group (green line) for females based on results shown in Table 4 (Vertical bars indicate standard deviation**.)

Figures 4 and 5 show differences in average CAVI scores by age between the CVD risk-free group and all CVD high-risk groups combined for men and for women, respectively. After 40 years of age, the difference in age-specific average CAVI scores became statistically significant between the two groups, with borderline significance in men 70-74 years of age (p = 0.054), and also became wider as age advanced both for men and for women.

## Discussion

As shown in Table 2, we have established the baseline CAVI scores based on 5,969 CVD risk-free persons selected out of 32,627 persons 20-74 years of age. It is shown that there exists a linear association between CAVI scores and age in both genders confirming that aging is an independent risk factor of atherosclerosis and cardiovascular disease as described in western [22,23] and Japanese studies [24]. Table 2 and Figure 3 show a biological aging of major arteries among CVD risk-free persons. We found that age-specific average CAVI scores among men were significantly greater than among women. Such a finding is consistent with the fact that men have a higher risk for coronary heart disease (of which one major risk factor is arteriosclerosis) than women [1,2]. Based on these findings, we need to evaluate an individual's CAVI scores according to his/her age and gender when we conduct screening.

As established by Framingham studies and others [25-27], hypertension is a risk factor of cardiovascular disease. Hypertension is also significantly associated with PWV [9,10,28]. High PWV values are found to be an independent predictive factor of cardiovascular disease [29]. Since our results indicate that age-specific average CAVI scores in the hypertension group were significantly higher than those from the CVD risk-free group (Tables 3, 4), it is implied that hypertension is a risk factor of arteriosclerosis.

The association between serum lipid levels and atherosclerotic disease, namely coronary heart disease, has been established through the findings from several epidemiological studies such as the Seven Countries Study [30], the Multiple Risk Factor Intervention Trial Study [31], and Klag et al's follow-up study [32]. Namekata et al. reported that abnormally high PWV was significantly associated with 4.5 or greater value of the ratio of total cholesterol to high density lipoprotein (HDL) cholesterol implying that abnormal lipid imbalance is a risk factor of arterial stiffness and arteriosclerosis [9]. Our results support such an association by showing that age-specific average CAVI scores among persons with hypercholesterolemia and hypertriglyceridemia of ages 40 and over were significantly greater than those among CVD risk-free persons for the same age-specific groups (Tables 3, 4).

Diabetes mellitus is proven to be a risk factor for cardiovascular disease [33,34]. It is reported that CVD risk among diabetics was 2-6 times higher than among non-diabetics and PWV values were associated with fasting glucose levels among diabetics [35,36]. An odds ratio for having abnormally high PWV among diabetics is also reported to be 3.66 (p < 0.001) as compared to non-diabetics [9]. Our results are consistent with these findings by showing significantly higher average age-specific CAVI scores among persons with hyperglycemia after 40 years of age than those among CVD risk-free persons (Tables 3, 4).

Ischemic changes in ECG and arteriolar changes in retina are considered as surrogate markers of arterial stiffness and arteriosclerosis in the coronary arteries and retinal arteries, respectively. It is also shown that atherosclerotic lesions in the aorta proceeds onset of CVD [37-39], as an increase in PWV values proceeds ischemic changes in ECG and arterial changes in retina appear [40]. We have shown that the age-specific average CAVI scores of the ischemic changes group and of the retinal artery changes group were significantly greater than those of the CVD risk-free group (Tables 3, 4). This implies that CAVI scores reflect the extent of arteriosclerotic changes not only in medium-size and large-size arteries but also in small-size arteries.

We have shown that age-specific average CAVI scores of all CVD high-risk persons combined were significantly higher than those of the CVD risk-free group after 40 years of age (Tables 3, 4), indicating that the overall arteriosclerosis status of the CVD high-risk group was significantly worse than that of the CVD risk-free group. Because no difference in average CAVI scores between the two groups was detected before 40 years of age, effective CAVI screening might be recommended for people age 40 and over.

With regard to the validity to use CAVI scores as an indicator of arteriosclerosis, Otsuka examined 72 deceased patients' ante-mortem PWV (which is a basis for deriving CAVI scores) and pathological changes measured by the diffuse fibrotic thickening, formation of atheroma and calcification in the wall of their aorta. He reported multiple regression coefficient R = 0.810 between PWV and scores of those pathological changes [41]. In addition, other researchers reported that CAVI scores were significantly associated with coronary atherosclerosis [13], with carotid intima-media thickness and with homocysteine [14]. Thus, the use of CAVI scores derived from PWV values is valid to estimate the extent of arteriosclerosis.

VaSera VS-1000, which was used in our study, was designed to measure CAVI scores independent of blood pressure and CAVI scores represent the extent of arteriosclerosis between the aortic valve and the ankle. We have shown biological aging of the major artery by measuring CAVI scores in the CVD risk-free group and disease-related pathological aging of the major artery in the CVD high-risk group. CAVI scores allow us to evaluate the extent of arteriosclerosis in the major arteries between the aortic valve and the ankle, to screen persons with subclinical stage of CVD, and provide an opportunity to modify diet and lifestyle to improve CAVI scores as reported by Satoh et al [42]. Thus, the use of CAVI scores potentially leads to savings on high treatment costs and to prolonging many productive lives.

There are some limitations in our study. First, the study design was cross-sectional and results were based on our observations at the time of screening. Secondly, our data did not include behavioral and lifestyle factors, although we consider that effects of such factors were reflected on clinical measurements related to CVD which we included. Currently we are examining the association between CAVI scores and lifestyle factors such as smoking, alcohol consumption, and body mass index, and will report results in the near future.

## Conclusions

Our results imply that advancement of arteriosclerosis among men is greater in every age group than among women. It is also implied that arteriosclerosis of the CVD high-risk group advances faster than that of the CVD risk-free group after 40 years of age. The baseline CAVI scores from the CVD risk-free group are useful for future studies as control values. The CAVI method is a useful tool to screen persons with moderate to advanced levels of arteriosclerosis.

## Competing interests

The authors declare that they have no competing interests.

## Authors' contributions

TN, KS and KS conceived and designed the study. KS and NI acquired the data. TN and KS performed statistical analyses. TN and KS drafted the manuscript, all other authors revised critically and approved the final manuscript.

## Pre-publication history

The pre-publication history for this paper can be accessed here:

http://www.biomedcentral.com/1471-2261/11/51/prepub
